# Epilepsy in Cerebrovascular Diseases: A Narrative Review

**DOI:** 10.2174/1570159X20666220706113925

**Published:** 2023-06-15

**Authors:** Sabrina Neri, Sara Gasparini, Angelo Pascarella, Domenico Santangelo, Vittoria Cianci, Anna Mammì, Michele Lo Giudice, Edoardo Ferlazzo, Umberto Aguglia

**Affiliations:** 1 Department of Medical and Surgical Sciences, Magna Graecia University, Catanzaro, Italy;; 2 Regional Epilepsy Centre, Great Metropolitan Hospital, Reggio Calabria, Italy

**Keywords:** Seizures, epilepsy, cerebrovascular disease, stroke, post-stroke epilepsy, cerebral haemorrhage, leukoaraiosis

## Abstract

***Background*:** Epilepsy is a common comorbidity of cerebrovascular disease and an increasing socioeconomic burden.

***Objective*:** We aimed to provide an updated comprehensive review on the state of the art about seizures and epilepsy in stroke, cerebral haemorrhage, and leukoaraiosis.

***Methods*:** We selected English-written articles on epilepsy, stroke, and small vessel disease up until December 2021. We reported the most recent data about epidemiology, pathophysiology, prognosis, and management for each disease.

***Results*:** The main predictors for both ES and PSE are the severity and extent of stroke, the presence of cortical involvement and hemorrhagic transformation, while PSE is also predicted by younger age at stroke onset. Few data exist on physiopathology and seizure semiology, and no randomized controlled trial has been performed to standardize the therapeutic approach to post-stroke epilepsy.

***Conclusion*:** Some aspects of ES and PSE have been well explored, particularly epidemiology and risk factors. On the contrary, few data exist on physiopathology, and existing evidence is mainly based on studies on animal models. Little is also known about seizure semiology, which may also be difficult to interpret by non-epileptologists. Moreover, the therapeutic approach needs standardization as regards indications and the choice of specific ASMs. Future research may help to better elucidate these aspects.

## 1 INTRODUCTION

The comorbidity between seizures or epilepsy and cerebrovascular disease is relevant and of particular interest nowadays. Cerebrovascular diseases are the pathogenetic factor that most frequently underlies epilepsy, especially in older patients [1, 2]. As the average age of the world's population increases, the number of elderly patients with both these conditions will rise.

Seizures are an important complication of stroke, as they increase mortality and morbidity and lengthen the period of hospitalization [2]. Seizures may appear in close association with the ischemic event or following a period of variable duration, comparably to what happens in post-traumatic epilepsy. Seizures occurring at the same time as the ischemic event or within seven days are termed acute symptomatic, provoked, situation-related, or early seizures (ES) [3], whereas those occurring after seven days of the stroke, are called late or remote symptomatic seizures [4]. These two entities differ in terms of both pathophysiologic mechanisms, short-term outcome and late reoccurrence risk.

The purpose of this article is to provide a comprehensive review of the clinical, epidemiologic, and therapeutic aspects of seizures and epilepsy associated with ischemic stroke, cerebral haemorrhage, reperfusion therapies (such as thrombolysis and thrombectomy), and small vessel disease due to arteriolosclerosis.

## 2 REVIEW OF THE LITERATURE

Medical publications concerning epilepsies and seizures in cerebrovascular diseases were reviewed. References were identified by searches of PubMed and Google Scholar until December 2021 with the terms “epilepsy” or “seizures” in various combinations with “stroke”, “cerebrovascular diseases”, “cerebral haemorrhage”, “leukoaraiosis”, “small vessel disease”, “reperfusion therapies”, “thrombolysis”, thrombectomy”. Articles were also identified through searches of the authors’ files. Selection criteria were novelty, importance, originality, quality and relevance to the scope of this review.

## 3 EARLY SEIZURES AFTER STROKE

### 3.1 Epidemiology and Clinical Features

The incidence and risk factors for the occurrence of ES after stroke have been extensively researched and described. The reported incidence of ES may vary widely depending on various factors such as the study design (retrospective, prospective, cohort or case/control), the method used to ascertain cases and also whether ischemic and hemorrhagic strokes are considered ad a single entity or separately. A prospective study of a cohort of patients with acute stroke (both hemorrhagic and ischemic), identified ES in 3.9% of patients [5], whereas a later meta-analysis conducted in 2017 to determine the incidence of ES after an ischemic stroke, concluded that they occurred in 3.3% of patients [6]. Another prospective study found a much lower frequency of ES for lacunar stroke (0,9%) [7]. The severity and extent of stroke, cortical involvement and its hemorrhagic transformation have been consistently and frequently associated with the risk of ES in various case series [5, 8-10]. Age, sex, some modifiable factors such as hypertension and diabetes, and the cause of the stroke do not seem to correlate in any way with the incidence of seizures [5, 7, 11]. Almost all ES occur within 24 to 48 hours of ischemia [9]. According to a multicenter prospective study of 2021 stroke patients followed for nine months, 43% had a seizure within the first 24 hours after stroke [9]. As for seizure semeiology, different case series found that post-stroke seizures usually appear to follow a localization-related semeiology and focal motor seizures with or without change in alertness account for approximately 50 to 90% of early post-stroke seizures [12, 13]. This finding probably reflects the higher incidence of strokes involving the middle cerebral artery territory [13]. Status epilepticus has been found to develop in 9-19% of cases [14, 15].

### 3.2 Pathophysiology

Immediately after a stroke, ES can occur through neuronal injury and transient cellular biochemical dysfunction [16]. The cellular events that are initiated by an acute blood flow deprivation caused by brain ischemia are similar to those happening when, during a seizure, neurons are maintained in a sustained depolarization state [17]. In animal models, stroke results in alterations in ion channels as early as one day after the insult. Damage to ion channels and increased intracellular sodium and calcium result in depolarization of the transmembrane potential and lowered seizures threshold. Moreover, the reduction of inhibitory GABAergic neurons in the cortex leads to neuronal instability and hyperexcitability [18, 19]. Excitatory amino acids like glutamate are massively released into the extracellular space, contributing to secondary neuronal damage by inappropriately activating AMPA and NMDA receptors and increasing sodium and calcium entry into neurons through the glutamate-gated ion channels [18, 20]. This last event initiates a cascade of cellular mechanisms that cause tissue damage, such as cell swelling, activation of proteolytic enzymes that degrade cytoskeletal proteins, generation of free radicals, impaired mitochondrial activity, and apoptosis [21].

### 3.3 Management

**Fig. (1):**
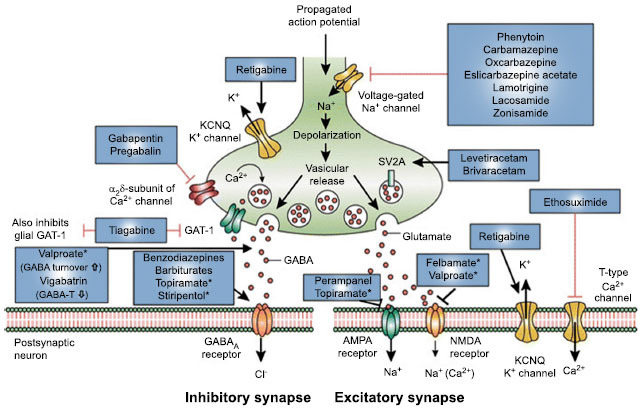
Mechanism of action of clinically approved anti-seizure drugs. Drugs marked with asterisks indicate that these compounds act by multiple mechanims (not all mechanisms shown here). GABA-T GABA aminotransferase, GAT GABA transporter, SV2A synaptic vesicle protein 2A, GABA gamma-aminobutyric acid, NMDA N-methyl-D-aspartate, AMPA α-amino-3-hydroxy-5-methyl-4-isoxazolepropionic acid, KCNQ a family of voltage-gated potassium channels (also known as the Kv7 family). Reprinted from CNS Drugs, 30, Löscher W, Gillard M, Sands ZA, Kaminski RM, Klitgaard H, Synaptic Vesicle Glycoprotein 2A Ligands in the Treatment of Epilepsy and Beyond, pages 1055-1077, Copyright @ 2016, with permission from Springer Nature. Reprints and Permissions.

There are no controlled studies evaluating the use of specific antiseizure medications (ASMs) in the treatment of ES, and the utility of initiating treatment after a single seizure is still debated. In the absence of clear guidelines, it is up to the individual clinician to decide when to treat early poststroke seizures [4]. A figure detailing the mechanism of action of clinically approved anti-seizure drugs is provided below (Fig. **1**). Post-stroke seizures usually respond to a single anticonvulsant [22]. There is no clear evidence neither on the relative efficacy of single ASMs for the treatment of ES, nor on the timing of ASM withdrawal after ES. Drugs with a parenteral formulation may be more advantageous because they can be administered in a patient with difficulty swallowing or disturbance of consciousness, as may occur after a stroke.

That stated, taking into account the cellular and tissue alterations described above that occur after a stroke, some animal studies have demonstrated the neuroprotective effect of some ASMs. Sodium ions are implicated in both seizure generation and mechanisms of acute neuronal death [18]. Phenytoin decreases brain excitability by reducing Na^+^ influx at pre and postsynaptic levels and has shown a neuroprotective effect in a cardiac arrest-induced global ischemia model [23]. Topiramate, in addition to its Na^+^ channel blocking activity, also can inhibit carbonic anhydrase, potentiate GABA response and impair AMPA/kainate glutamate receptor activity while also opening potassium channels [24]. Topiramate reduced the degree of motor impairment and severity of seizures induced by global ischemia in rat models. The highest dose of topiramate prevented most of the histological signs of hippocampal ischemic neuronal injury [25].

Another Na+ channel blocker, zonisamide, which is extensively used as a broad-spectrum ASM, has been shown, in rat models of focal cerebral ischemia caused by middle cerebral artery occlusion, to scavenge hydroxyl radicals and nitric oxide in a dose-dependent manner, therefore probably protecting neurons from free radical damage and stabilization of neuronal membranes [26].

ASMs acting as calcium antagonists do not reduce the risk of death after acute stroke but might have a critical role in the “delayed” neuronal death due to increased intracellular Ca^+^ [27]. Lamotrigine, administered immediately (within 2 hours) after the occlusion of MCA in rats, reduced the infarct volume and the neurological deficits assessed 24 h after the stroke [28]. In another study, this finding was not confirmed [29].

The increase in GABA-mediated neurotransmission, as it opposes excitatory glutamatergic activity, might have a neuroprotective effect [30]. Diazepam, a positive allosteric modulator of *GABA* A receptors, protected CA1 hippocampal pyramidal cells in gerbil models of transient cerebral ischemia [31]. While a large body of evidence exists that supports the neuroprotective action of ASMs in animal models of ischemia, the same evidence is lacking in clinical studies. This could be due to clinical designs and limited sensitivity of functional outcome measures in humans, but also to intrinsic differences between animals and humans.

### 3.4 Outcome and Prognosis

Patients with single ES have a risk of long-term seizures recurrence of approximately 30% [32] over the following 10 years. This risk is increased fourfold as compared to patients who do not experience ES [4]. In a hospital-based stroke cohort, early status epilepticus was not associated with a further increase in the risk of remote seizures [14]. The effect of ES on stroke outcomes is still debated. Some studies have reported higher long-term mortality/disability [10, 12] while other population-based studies did not find an association between ES and stroke outcome [7] deeming the presence of ES as just a correlate of stroke severity. As for why ES should worsen the outcome of stroke, it can be assumed that ES in the ischemic penumbra adds metabolic stress to an already vulnerable tissue [33].

## 4 LATE SEIZURES

Seizures occurring after at least seven days following a stroke, are called late or remote symptomatic seizures. Following the 2014 ILAE epilepsy definition [34], the term post-stroke epilepsy (PSE) can be also used. According to the definition, PSE may be diagnosed after a single seizure if a high risk of recurrence exists. The risk increases with remote structural lesions, such as those occurring after a stroke [34]. In 34 longitudinal cohort studies, which analyzed more than 100,000 patients, the incidence of PSE was 7% [35], representing 30-49% of all new-onset seizures in patients aged >65 years [36]. The risk of 10-year recurrence after a first unprovoked seizure is >70% [37].

In a large, long-term population-based study, the highest risk of having a first unprovoked seizure occurred in the first year of follow-up [38]. Another study found that the annual incidence of PSE after the first stroke was 6.3% after 1 year, 2.4% after two, 1.3% after three, and 0.3% per year in the following years [39].

It is possible that, especially in subjects with poorer functional outcomes after stroke, some non-motor focal seizures might not be identified. Some seizures, for example, those with subsequent Todd's palsy, may erroneously lead to the diagnosis of recurrent stroke [40]. Moreover, not all post-stroke seizures are caused by stroke, but, especially in an elderly patient, they could be due to other neurological (central nervous system infections) or metabolic (electrolyte imbalances or sepsis) causes. Thus, the actual prevalence of PSE may be wrongly estimated.

### 4.1 Predictors of PSE

Several cohort studies agree on the clinical predictors of PSE. Younger age at stroke onset appears to be predictive of PSE. Specifically, in a study on 3000 stroke patients, subjects younger than 65 years had a higher risk of PSE than those over 85 years old [38]. Several other studies agree with this finding [41-43]. In opposition, in a population-based study performed in Rochester, Minnesota, the absolute risk of PSE was similar in patients younger than 55 years and older than 75 years [44]. It should be kept in mind, however, that the incidence of seizures in the elderly patient could be underestimated for several reasons, including the presence of non-convulsive seizures that are difficult to identify in a subject with multiple pathologies and, possibly, cognitive decline; the more thorough diagnostic approach a young patient with stroke is subjected to, which might prompt to easily recognize the presence of seizures; and lastly the higher survival rates of young people after a stroke [45].

Large infarcts involving the territories of the internal carotid or middle cerebral artery are associated with an increased risk of PSE [38, 46] with a higher incidence when compared to microangiopathic or cardioembolic stroke etiologies [41]. Regardless of the involved artery, damage to cortical areas predicts the occurrence of PSE [18]. A metanalysis found that cortical involvement is an independent factor for PSE, causing a fourfold increase in risk [4]. Higher National Institutes of Health Stroke Scale (NIHSS) scores, which usually correlate with severe strokes involving broad cortical areas, are significantly associated with the risk for PSE [41]. In the Oxfordshire Community Stroke Project published in 1997, only 1% of patients with lacunar strokes developed PSE, while 11% of subjects with TACI experienced late-onset seizures [10]. Subsequent studies suggest the same finding, with patients with small lesions that very rarely develop seizures [18, 42].

Equally to ES, the presence of a hemorrhagic component (either primary or as a transformation of ischemic stroke) highly predicts PSE [42, 45] and the risk of PSE after a hemorrhagic stroke is almost doubled [4].

In a retrospective study on 875 patients surviving at least seven days after an intracerebral hemorrhage, 8.1% developed ES and 9.8% late seizures. The risk of developing drug-resistant epilepsy was higher in patients who experienced ES. This finding could be of importance in deciding the therapeutic strategies to minimize the risk of developing drug-resistant post-hemorrhage epilepsy [47].

Seizures after a haemorrhage are thought to result from irritation of the surviving parenchyma by-products of blood metabolism [48]. Indeed, hemosiderin is thought to irritate neurons, as administration of iron in mice causes focal epilepsy [49]. Moreover, electrophysiological studies carried out in the brain parenchyma adjacent to the hemorrhagic lesion have shown the possibility of spontaneous synaptic activity and increased excitability of these neurons [50]. The location of blood is also an important predictor of PSE. A prospective study of 123 patients identified an incidence of seizures at 54% in the case of lobar cortical haemorrhage, 19% in the case of haemorrhage in the basal ganglia, and 0% in the case of thalamic haemorrhage [51]. Based on the Helsinki population, Hapaniemi's group developed the CAVE score to predict the development of late seizures after a primary cerebral haemorrhage and stratify risk. The score associates one point with each of the following items: cortical involvement, age under 65 years, blood volume greater than 10 mL, and ES within seven days of the event. The score ranges from 0 to 4 and the cumulative seizure risk ranges from 0.6% to 46.2%. This score is yet to be validated for the treatment of cerebral haemorrhage survivors [52].

The family history of seizures has been found to strongly correlate with the occurrence of PSE [8]. However, the implication of gene variants in the risk of PSE has only been investigated by two studies, which demonstrated that polymorphisms in mitochondrial aldehyde dehydrogenase 2 (ALDH2) [53] and CD40 protein [54], respectively, are associated with a higher risk of PSE in carriers. Further studies aimed at better delineating the relationship between gene variants and PSE risk are needed.

Some authors investigated the radiological predictors of post-stroke epilepsy. Similar to what was stated above, larger lesions and cortical involvement in imaging are independent factors for seizures occurrence [55]. In particular, involvement of the parietal-temporal cortex, supramarginal gyrus, and superior temporal gyrus seems to predispose more to PSE [8, 9, 56].

One study investigated the possible use of the Alberta Stroke Program Early CT (ASPECT) score as a screening to identify patients at risk of developing PSE [57]. The ASPECT score correlates inversely with the severity and extent of ischemic injury. The authors concluded that low ASPECT scores, performed on admission and after 24 hours, were associated with a higher incidence of seizures [57]. Other multivariate analyses found cortical involvement and large lesion sites to be independent predictors of epileptic seizures [58, 59].

A score based on 1200 stroke subjects, called the SeLECT score, was developed to be a prognostic model of post-stroke epilepsy [60]. It is based on five parameters: severity of stroke, assessed by NIHSS (score 0-2 points), large artery atherosclerotic aetiology (0-1 points), ES (0-3 points), cortical involvement (0-1 points) and middle cerebral artery involvement (0-1 point). The lowest SeLECT score (0 points) was associated with a 0,7% risk of late seizures within one year after stroke and 1.3% within 5 years, whereas the highest value (9 points) predicted a 63% risk of late seizures within 1 year and 83% risk within 5 years [60].

Regarding possible electroencephalographic predictors of seizures, in a cohort of 151 subjects with stroke who underwent video EEG in the first 72 h after a stroke, asymmetry of background activity and interictal epileptiform discharges were independently associated with a 3-fold risk of PSE [61]. Moreover, a retrospective analysis of a large number of stroke patients identified rhythmic intermittent frontal delta activity (FIRDA) and diffuse slowing as being related to an increased risk of PSE [62]. Other authors, in contrast, deny the correlation between early EEG abnormalities after stroke and the development of PSE [63]. The interpretation of electroencephalographic findings in patients with stroke may be challenging and could lead to misdiagnosis because some EEG alterations, such as focal slowing, are often identified in patients with stroke without seizures [12].

Reperfusion therapy using thrombolysis with the administration of recombinant tissue plasminogen activator (rt-PA: intravenous alteplase) and endovascular interventions such as mechanical thrombectomy (MT) are current, highly effective treatments for the treatment of cerebral ischemia. The epileptogenic and neurotoxic role of tissue plasminogen activator (t-PA) has been demonstrated in animal models: t-PA expression has been found to increase after seizures [64] and mice with a deficiency in endogenous tissue plasminogen activator are found to be less prone to chemo convulsant-induced seizures [65].

To date, conflicting evidence exists regarding the risk of seizures and epilepsy post-reperfusion therapy. A meta-analysis published in 2019 by Gasparini and colleagues, which sought to identify risk factors for post-stroke epilepsy, found that while young age, cortical involvement, hemorrhagic transformation of the lesion, and ES predisposed to PSE, sex and rt-PA treatment did not predict the occurrence of it [43]. A subsequent case-control study performed in a single stroke centre retrospectively collected cases of patients with seizures occurring in the first seven days after stroke and identified that thrombolysis appeared to be an independent risk factor for the development of ES [66]. Regarding data on patients treated exclusively with thrombectomy, existing studies to date agree on a low incidence of both ES [67, 68] and PSE [69].

### 4.2 Mechanisms of PSE

The epileptogenic mechanisms of PSE differ from that of ES and are associated with chronic, persistent changes that result from ischemic damage. At the moment, the most accredited mechanisms are believed to be: the blood-brain barrier (BBB) dysfunction, the organization of an epileptogenic glial scar, the alteration of ion channels. Underlying each of these mechanisms, the role of epigenetics could also be speculated.

The role of BBB dysfunction in the genesis of PSE was highlighted in a small cohort of 28 patients with cortical stroke who developed PSE approximately 1 year after the ischemic event [70]. These patients underwent Single Photon Emission Computed Tomography (SPECT) imaging with ^99m^Tc-diethylene within 72 hours after the last seizure, and in 86% of patients, SPECT revealed alteration of the BBB at the site of the previous cortical stroke. In the control group with stroke without seizure, 29% had BBB alteration in the region of the cortical stroke at SPECT. The reason why not all patients with BBB went on to seizures was not clarified by the authors. However, it is not at all clear whether the relationship between the BBB disruption and PSE is bidirectional. Indeed, it is arguable that BBB alteration may function as both a cause and a consequence of epilepsy [18].

Alteration of BBB function is well known to occur during status epilepticus and refractory epilepsy, particularly of the temporal lobe [71]. However, it is also closely associated with stroke damage [72]. As a result of BBB dysfunction, a cascade of subsequent events occurs, leading to neuronal hyperexcitability and hype-synchronization [73]. Provoked seizures further damage the integrity of the blood-brain barrier, with the release of proinflammatory cytokines, which further reduces the seizure threshold [73]. BBB dysfunction, vasogenic oedema, glutamate toxicity, and altered cell ion gradients may also contribute to secondary and persistent brain damage (gliotic scarring and leukoaraiosis) [18, 74], the role of which in the generation of PSE will be discussed below.

The replacement of the necrotic area with microglia and the formation of the gliotic scar is a well-known event after a stroke [75], but reactive astrocytes are also commonly found in epilepsy patients, such as those with hippocampal sclerosis [76]. Despite the undeniable role of astrocytes as a scaffold for angiogenesis and nerve regrowth, astrogliosis and glial scar formation are accompanied by diverse biological and physiological consequences that might promote neuronal excitability. These changes include also impaired glutamate uptake, which causes elevated glutamate and potassium levels in the extracellular space and therefore hyperexcitability in near neurons. Reactive astrocytes also have impaired glutamine synthetase function, and the reduced conversion of glutamate into glutamine reduces GABA synthesis in inhibitory interneurons [77].

Lastly, stroke alters calcium and sodium channels in mouse models in a short period of time [78]. Ion channel damage alters neuronal firing increasing their excitatory function, and increased intercellular calcium and sodium lower critical threshold for depolarization [18].

The common mechanisms for these alterations might be represented by changes in gene expression that start to occur immediately after the onset of the stroke. This has been demonstrated in mouse models of middle cerebral artery occlusion [79]. Liu and colleagues studied the time-specific pattern of alterations in gene expression following a hemispheric injury after transient middle cerebral artery occlusion. They found that post-stroke transcriptional changes occur in a dynamic and coordinated manner as early as 30 minutes after the ischemic event, starting with modifications in the transcription of heat-shock proteins and culminating in alterations in neuronal plasticity and immune response several days after the event [79]. Furthermore, miRNA (which are short non-coding RNAs involved in mRNA degradation) expression in cortical ischemic tissue also changes between 7 and 14 days after stroke, suggesting that miRNAs participate in parenchyma damage, neuroplasticity, glial scar formation, and regulation of neuronal excitability [80]. Together, these events act in a complexly intricate manner and, by causing structural and functional alterations, likely contribute to persistent epileptogenesis.

### 4.3 Clinical Manifestations of PSE

PSE seizures tend to occur primarily as focal seizures, with or without evolution to bilateral tonic-clonic [12, 81]. Regarding status epilepticus, in a large case series of patients with post-stroke seizures, 17 patients (9%) presented with status epilepticus, 10 of whom >7 days after the stroke. There was no relationship between the occurrence of SE and cerebrovascular risk factors, stroke type (ischemic or hemorrhagic), topography and cause, cortical involvement, size of lesion, seizure type, or electroencephalographic findings. SE occurred more frequently among patients with a higher functional disability after the acute phase of stroke calculated on the Rankin scale [15].

### 4.4 Management

Because PSE primarily affects older patients, the choice of the most appropriate antiseizure medication (ASM) should always consider each patient's characteristics, including any comorbidities and concomitant therapies.

Older patients are also more prone to adverse drug events, thus requiring slower titration and possibly lower doses to reach the therapeutic range [4]. For these reasons, second-generation ASMs are certainly better suited because of their fewer side effects and safer pharmacokinetic profiles.

PSE has an excellent prognosis in terms of seizure control, with different observational and prospective studies reporting a high number of seizure-free patients (54%-67% [82, 83]) even in monotherapy. Despite this, a proportion of patients present with drug-resistant epilepsy. In a retrospective cohort of patients with post-stroke epilepsy, 18.2% were drug-resistant. Younger age at stroke onset, hemorrhagic stroke, stroke severity, status epilepticus as presenting symptom and focal to bilateral tonic-clonic seizures were independently associated with drug resistance [84]. In another study, the male sex was associated with a lower risk of drug-resistant epilepsy in stroke patients aged 67 and over [85].

Drug resistance is likely the result of multiple mechanisms. One of those is represented by changes in ion channels and neurotransmitter receptors' distribution and function. A reduction in the distribution of GABA A receptor subunits has been shown in animal models of ischemic stroke both in the regions surrounding a brain lesion and in remote cerebral tissue. This alteration may fail some classes of anti-seizure drugs, particularly those that act on GABA-ergic pathways [86]. Post-stroke plasticity with aberrant synaptogenesis and the generation of hypersincronized networks which favour seizure generation might also restrict ASMs from accessing neural targets [87].

Few data are available on the relative efficacy of single ASMs, as no randomized controlled trial has been performed specifically on PSE. To date, only two randomized controlled trials have specifically evaluated AEDs in post-stroke seizures and they are included in a systematic review and meta-analysis [88]. A table reporting the main studies evaluating the use of ASMs in PSE is provided (Table **1**).

Several trials have identified carbamazepine (CBZ) [89], lamotrigine (LTG) [90], and levetiracetam (LEV) [91] as useful ASMs in patients with late-onset epilepsy, including PSE. In a prospective observational study [91] conducted on 35 patients with late-onset post-stroke seizures, 77.1% of patients achieved 1-year seizure freedom. Drug withdrawal due to side effects occurred in 11.4% of patients. In another prospective study [92] on 34 patients, 82.4% of them achieved seizure freedom at 1000-2000 mg of LEV per day, whereas 20.6% experienced side effects. Therefore LEV seems to be an effective and well-tolerated option as monotherapy against post-stroke seizures.

A randomized, double-blind trial comparing CBZ, LTG, and LEV 359 elderly patients with new-onset focal epilepsy, many with cerebrovascular lesions, identified LTG and LEV as better tolerated than CBZ with a retention rate of respectively 61.5%, 55.6% and 45.8% at 58 weeks [93]. But when comparing indirectly LEV and LTG, using controlled release-CBZ as a comparator, LTG has been found to associate with fewer side effects than LEV and a lower rate of drug withdrawal [90].

A large cohort study [94] showed that LTG was associated with a lower mortality rate than CBZ in patients with PSE. LEV was associated with a reduced risk of cardiovascular death compared with CBZ, without a difference in overall mortality.

Regarding the use of ASMs in primary prevention, no evidence demonstrates their usefulness in preventing ES nor PSE [4], and the European Stroke Organization guidelines for the management of post-stroke seizures and epilepsy do not recommend immediate primary prophylaxis with an ASM for adults with ischemic stroke or intracranial hemorrhage [95]. Conversely, different animal studies have shown that statins, competitive inhibitors of hydroxymethylglutaryl (HMG) CoA reductase, have a neuroprotective role [96]. Statins can reduce excitotoxicity by reducing glutamate uptake, the expression of anti-apoptotic proteins, suppression of astrogliosis, and reducing blood-brain barrier permeability [97, 98]. A systematic review on the use of statins for primary prevention of seizures after stroke showed that statins have indeed a protective effect on PSE after ischemic stroke and ES and PSE after hemorrhagic stroke [99]. However, there are some concerns over the use of high doses of statins for PSE prevention, as an increased risk of intracerebral hemorrhage was reported [100]. Moreover, statins are metabolized by the cytochrome 3A4 system, therefore their serum levels can be reduced by the concomitant use of enzyme-inducing ASMs [101]. Thus, despite these promising data, there is insufficient evidence to suggest the use of statins as a preventative treatment for post-stroke epilepsy.

## 5 SEIZURES IN LEUKOARAIOSIS OR SMALL VESSEL DISEASE

Leukoaraiosis is a pathology of white matter due to hypoxia-ischemia caused by disease of the small vessels, predominantly the perforating arteries, capillaries, and venules [74]. Leukoaraiosis is a common finding in patients with vascular risk factors, particularly hypertension [102] and its prevalence increases with age, ranging from less than 20% in people aged less than 55 years to more than 80% in those aged more than 85 years [103]. The severity of leukoaraiosis also tends to increase with age [102].

Leukoaraiosis, even in the absence of a manifest stroke, has been associated with the development of epilepsy. Studies in animal models have demonstrated small vessel involvement in epileptogenesis, with mice with leukoaraiosis exhibiting temporal lobe epilepsy [104] and a higher predisposition to the development of kainate-induced status epilepticus in comparison to control rats [105].

Although considered uncommon by some authors, the association between seizures and leukoaraiosis [106, 107] has also been reported in several clinical studies. For example, a retrospective study of 223 patients with leukoaraiosis described that seizures occurred in 24% of patients, especially with frontal or parieto-occipital lesions. The authors speculated that U-fiber involvement may increase the propensity for seizures [108].

Another study on patients with cryptogenic late-onset epilepsy demonstrated a greater presence of leukoaraiosis in cases than in controls. Unfortunately, half of the patients in this series did not undergo brain MRI, thus limiting the possibility to generalize and validate the results [109].

A retrospective study on 117 patients who underwent MRI described the type of epilepsy associated with stroke and leukoaraiosis, respectively. The two groups (patients with stroke-associated epilepsy and patients with leukoaraiosis-associated epilepsy) differed in the location of seizure onset: in patients with PSE, the epileptogenic focus was consistent with the previous vascular lesion, whereas patients with leukoaraiosis-associated epilepsy had a higher rate of temporal lobe epilepsy. Thus, the authors hypothesized that temporal lobe structures may be more susceptible to seizure development in the setting of leukoaraiosis [110]. Because temporal lobe epilepsy is the most common type of focal epilepsy, there may be a casual association between it and leukoaraiosis [111].

In addition, leukoaraiosis may cause cortical microinfarcts visible only to the most advanced imaging techniques [112], which are not currently used in clinical practice and were not used in the studies mentioned above. Consequently, it could be this possible variable that is actually responsible for the incidence of seizures in patients with leukoaraiosis. Higher field MRI (such as 7T) is a promising tool to use in subsequent clinical studies on leukoaraiosis and its involvement in epilepsy.

Among the risk factors for small vessel disease, hypertension is one of the most significant. In this context, it indirectly causes epilepsy because it contributes to the genesis of potentially epileptogenic parenchymal lesions. Moreover, hypertension can also act with a direct mechanism (not mediated by the presence of leukoaraiosis). In this context, hypertension becomes an independent risk factor for epilepsy, as shown in different studies [113, 114]. The pathogenic mechanism is still poorly investigated. Different animal models of epilepsy show either upregulation of the renin-angiotensin system in the hippocampus [115], or delayed seizure onset and reduced seizure frequency in rats with comorbid epilepsy and hypertension treated with anti-hypertensive drugs [116]. AT1 and AT2 receptors have also been found to be upregulated in the hippocampus of patients who underwent temporal lobectomy for drug-resistant temporal lobe epilepsy [117]. Therefore, angiotensin might act as a neurotransmitter/neuromodulator in different cerebral pathways and hypertension might represent a predictor of late-onset epilepsy independently from vascular damage.

## CONCLUSION

Epilepsy associated with cerebrovascular disease represents a common issue in the elderly population. Its prevalence will certainly grow in the next years, due to the increasing mean population age and longer survival after stroke. Some aspects of ES and PSE have been well explored, particularly epidemiology and risk factors. The main predictors for both ES and PSE are the severity and extent of stroke, the presence of cortical involvement, and haemorrhagic transformation, while PSE is also predicted by younger age at stroke onset.

On the contrary, few data exist on physiopathology, and existing evidence is mainly based on studies on animal models. From a clinical point of view, little is known about seizure semiology, which may also be difficult to interpret by non-epileptologists. Moreover, the therapeutic approach needs standardization as regards indications and the choice of specific ASMs. Future research may help to better elucidate these aspects.

## Figures and Tables

**Table 1:** Summary of the studies evaluating the use of ASMs in patients with PSE.

**References**	**Type of Study**	**No. of Patients**	**ASMs Evaluated**	**Findings**
Alvarez-Sabin *et al.*, 2002 [118]	Prospective, observational	71	GBP	Low efficacy and adverse events
Gilad *et al.*, 2007 [90]	Randomized, open label	64	LTG *vs*. sustained release CBZ	Both effective, LTG better tolerated
Kutlu *et al.*, 2008 [92]	Uncontrolled, open label	34	LEV	Effective and well tolerated
Belcastro *et al.*, 2008 [91]	Uncontrolled, open label	35	LEV	Effective and well tolerated
Consoli *et al.*, 2012 [119]	Randomized, open label	106	LEV *vs*. sustained release CBZ	Both effective, LEV better tolerated
Huang *et al.*, 2015 [120]	Retrospective	3622	PHT, CBZ, VPA *vs.* other AEDs (OXC, VGB, TGB, LTG, TPM, GBP, LEV, PRG)	VPA and other AEDs (OXC, VGB, TGB, LTG, TPM, GBP, LEV, PRG) higher efficacy than PHT
Tanaka *et al.*, 2017 [18]	Retrospective, observational	112	VPA, PHT, CBZ	Low tolerability, interaction with other drugs
Sales *et al.* 2020 [121]	Prospective, observational	1666	ESL	Effective and well tolerated, but interaction with other drugs

**Abbreviations:** CBZ: carbamazepine; ESL: eslicarbazepine; GBP: gabapentin; LEV: levetiracetam; LTG: lamotrigine; OXC: oxcarbazepine; PHT: phenytoin; PRG: pregabalin; TGB: tiagabine; TPM: topiramate; VPA: valproic acid; VGB: vigabatrin.

## LIST OF ABBREVIATIONS

ALDH2: Aldehyde Dehydrogenase 2
ASMs: Antiseizure Medications
ASPECT: Alberta Stroke Program Early CT
CBZ: Carbamazepine
ES: Early Seizures
LEV: Levetiracetam
LTG: Lamotrigine
MT: Mechanical Thrombectomy
NIHSS: National Institutes of Health Stroke Scale
PSE: Post-stroke Epilepsy
SPECT: Single Photon Emission Computed Tomography
